# An Observational Cohort Study on the Incidence of Severe Acute Respiratory Syndrome Coronavirus 2 (SARS-CoV-2) Infection and B.1.1.7 Variant Infection in Healthcare Workers by Antibody and Vaccination Status

**DOI:** 10.1093/cid/ciab608

**Published:** 2021-07-03

**Authors:** Sheila F Lumley, Gillian Rodger, Bede Constantinides, Nicholas Sanderson, Kevin K Chau, Teresa L Street, Denise O’Donnell, Alison Howarth, Stephanie B Hatch, Brian D Marsden, Stuart Cox, Tim James, Fiona Warren, Liam J Peck, Thomas G Ritter, Zoe de Toledo, Laura Warren, David Axten, Richard J Cornall, E Yvonne Jones, David I Stuart, Gavin Screaton, Daniel Ebner, Sarah Hoosdally, Meera Chand, Derrick W Crook, Anne-Marie O’Donnell, Christopher P Conlon, Koen B Pouwels, A Sarah Walker, Tim E A Peto, Susan Hopkins, Timothy M Walker, Nicole E Stoesser, Philippa C Matthews, Katie Jeffery, David W Eyre, Oxford University Hospitals Staff Testing Group, Afrah Shibu, Aisling Curtis, Alexandra Mighiu, Ali Manji, Andrey Nezhentsev, Arun Somanathan, Beinn Khulusi, Ben Holloway, Caitlin Rigler, Charis Virgo, Charlotte Fields, Charlotte Lee, Elizabeth Daly, Elizabeth Hatton, Esme Weeks, Euan McGivern, Greta Economides, Hannah Fuchs, Harry Jackson-Smith, Heather Tong, Helen Callard, Helen Clay, Henrietta Davies, Isaac Jarratt Barnham, Ishta Sharma, Jack Wilson, Jocelyn Ward, Joseph Cutteridge, Julia Kotowska, Kirsten Lee, Krupa Ravi, Laura Wilkins, Lottie Cansdale, Lucy Bland, Luiza Farache Trajano, Magdalena Chmura, Maria Lucey, Maria Pikoula, Meirian Evans, Molly Abbott, Morwenna Tamblyn, Oriane Grant, Rebecca Conway-Jones, Ross Toward, Roxanna Abhari, Ruby Wolman, Sara Hosseinzadeh, Sarah Thomas, Tara Madsen, Thomas H Foord, Thomas Johnson, Vimukthi Perera, Zamin Shabir, Thomas Christott, George Doherty, Philip W Fowler, Fredrik Karpe, James Kavanagh, Lucas Martins Ferreira, Matt J Neville, Hayleah Pickford, Donal Skelly, Jeremy Swann, Sarah Cameron, Phoebe Tamblin-Hopper, Magda Wolna, Rachael Brown, Denis Volk, Fan Yang-Turner, Alison Vaughan, Adrian Bialek, Alison Whitty, Annie Westlake, Barbara Wozniak, Bryony Butler, Claudio Ferreira, Danielle Russell, Dawn Pether, Elaine Lawson, Eleanor Ross, Eleni Fragkouli, Elizabeth Sims, Emma Mortimore, Geraldine Shaw, Harriet Mullins, Harriett Caroll, Jane Phillips, Jenny Brown, Jess Ponting, Justyna Szczurkowska, Kim Vilca, Kitty Norris, Louise Holland, Michael Luciw, Michelle Gates, Michelle Layton, Nicola Antonucci, Noemi Bodo, Rebecca Millard, Sara Lyden, Sarah Young, Simran Barot, Vanessa Cox, Victoria Wharton, Zoe Thompson, Anne Baby, Jasmine Bastable, Kathryn Cann, Reena Chohan, Josie Clarke, Gabriel Cogorno, Samantha Cordy, Georgina Coward, David Crawford-Jones, Sean Crawley, Jack Dobson, Bronte Drummond, Laura Dunn, Caleb Edwin, Simon Evans, Mohamad Fadzillah, Jessica Gentry, Sarah Hill, Laura Hobden, Nurul Huda, Gemma Innes, Scott Jarvis, Gerald Jesuthasan, Emma Jones, Anita Justice, Elizabeth Kalimeris, Richard Kirton, Nakiah Lashley, Sophie Mason, Alexander Mobbs, Ahila Murugathasan, Eleanor Mustoe, Gospel Ngoke, Sarah Oakley, Oliver O’Sullivan, Kimberley Odwin, Jack Oliver, Freyja Pattrick, Claudia Pereira, Simon Perry, Tom Potter, Alexander Prentice, Sophie Ramage, Athena Sanders, Kellyanne Savage, Katherine Shimell, Robin Terry, Emma Thornton, Susan Wareing, Annie Welbourne, Maddison Wheatley, Lisa Butcher, Gabriella D’Amato, Ruth Moroney, Gemma Pill, Lydia Rylance-Knight, Claire Sutton, Claudia Salvagno, Merline Tabirao, Sarah Wright

**Affiliations:** 1 Oxford University Hospitals NHS Foundation Trust, Oxford, United Kingdom; 2 Nuffield Department of Medicine, University of Oxford, Oxford, United Kingdom; 3 NIHR Oxford Biomedical Research Centre, University of Oxford, Oxford, United Kingdom; 4 NIHR Health Protection Research Unit in Healthcare Associated Infections and Antimicrobial Resistance at University of Oxford in partnership with Public Health England, Oxford, United Kingdom; 5 Kennedy Institute of Rheumatology Research, University of Oxford, United Kingdom; 6 Medical School, University of Oxford, Oxford, United Kingdom; 7 Target Discovery Institute, University of Oxford, Oxford, United Kingdom; 8 National Infection Service, Public Health England Colindale, United Kingdom; 9 Nuffield Department of Population Health, University of Oxford, Oxford, United Kingdom; 10 Oxford University Clinical Research Unit, Ho Chi Minh City, Vietnam; 11 Big Data Institute, University of Oxford, Oxford, United Kingdom

**Keywords:** SARS-CoV-2, vaccine, antibody, healthcare worker, immunity

## Abstract

**Background:**

Natural and vaccine-induced immunity will play a key role in controlling the severe acute respiratory syndrome coronavirus 2 (SARS-CoV-2) pandemic. SARS-CoV-2 variants have the potential to evade natural and vaccine-induced immunity.

**Methods:**

In a longitudinal cohort study of healthcare workers (HCWs) in Oxfordshire, United Kingdom, we investigated the protection from symptomatic and asymptomatic polymerase chain reaction (PCR)-confirmed SARS-CoV-2 infection conferred by vaccination (Pfizer-BioNTech BNT162b2, Oxford-AstraZeneca ChAdOx1 nCOV-19) and prior infection (determined using anti-spike antibody status), using Poisson regression adjusted for age, sex, temporal changes in incidence and role. We estimated protection conferred after 1 versus 2 vaccinations and from infections with the B.1.1.7 variant identified using whole genome sequencing.

**Results:**

In total, 13 109 HCWs participated; 8285 received the Pfizer-BioNTech vaccine (1407 two doses), and 2738 the Oxford-AstraZeneca vaccine (49 two doses). Compared to unvaccinated seronegative HCWs, natural immunity and 2 vaccination doses provided similar protection against symptomatic infection: no HCW vaccinated twice had symptomatic infection, and incidence was 98% lower in seropositive HCWs (adjusted incidence rate ratio 0.02 [95% confidence interval {CI} < .01–.18]). Two vaccine doses or seropositivity reduced the incidence of any PCR-positive result with or without symptoms by 90% (0.10 [95% CI .02–.38]) and 85% (0.15 [95% CI .08–.26]), respectively. Single-dose vaccination reduced the incidence of symptomatic infection by 67% (0.33 [95% CI .21–.52]) and any PCR-positive result by 64% (0.36 [95% CI .26–.50]). There was no evidence of differences in immunity induced by natural infection and vaccination for infections with S-gene target failure and B.1.1.7.

**Conclusions:**

Natural infection resulting in detectable anti-spike antibodies and 2 vaccine doses both provide robust protection against SARS-CoV-2 infection, including against the B.1.1.7 variant.

## Teaser

Natural infection resulting in detectable anti-spike antibodies and two vaccine doses both provided ≥85% protection against symptomatic and asymptomatic SARS-CoV-2 infection in healthcare workers, including against the B.1.1.7 variant. Single dose vaccination reduced symptomatic infection by 67%.

The severe acute respiratory syndrome coronavirus 2 (SARS-CoV-2) pandemic has had a global impact on morbidity and mortality [1]. Natural and vaccine-induced immunity will play a key role in controlling the pandemic, by reducing transmission, hospitalization and mortality. However, the ability of new SARS-CoV-2 variants to evade natural and vaccine-induced immunity mounted against ancestral viruses is of major public health concern.

Prior SARS-CoV-2 infection protects against polymerase chain reaction (PCR)-confirmed symptomatic/asymptomatic SARS-CoV-2 infection by 83–88% up to 5–6 months postinfection, with greater reductions in symptomatic reinfections [2–4]. Ongoing longitudinal studies are required to determine the duration of protection conferred by natural immunity; however evaluating this will be more difficult with widespread vaccination. Understanding the interaction between prior infection/serostatus and vaccination on protection from infection is also important.

Three vaccines have been approved for use in the United Kingdom to date [5], with Pfizer-BioNTech BNT162b2 and Oxford-AstraZeneca ChAdOx1 nCoV-19 (AZD1222) currently the most widely deployed, with many individuals receiving only one dose to date following a government decision to extend the dosing interval to 12 weeks to maximize initial coverage. For BNT162b2, trials demonstrated 95% efficacy in preventing symptomatic PCR-confirmed infection >7 days post-second dose; these findings have been replicated in several real-world studies including in Israel (92% effectiveness) [6] and the United Kingdom (88% effectiveness in individuals >80 years [7]; 85% reduction in all-PCR positives in a cohort of healthcare workers [HCWs]) [8]. Vaccine efficacy of 50–90% is seen following a single dose, dependent on population demographics, exposures and time-frame studied [6, 7, 9–13]. Fewer real-world data are available for ChAdOx1 nCoV-19, due to its later regulatory approval. Trials demonstrated vaccine efficacy of 62% against PCR-positive infection >14 days post-second dose using a standard dose/standard dose regimen, with subsequent analysis showing a higher efficacy of 81% in those with a longer dosing interval (>12 weeks). Single dose vaccine efficacy >22 days post-first dose has been reported as 69–76% [14, 15]. No real-world data on vaccine effectiveness against PCR-positive infections has been published, but preliminary analyses show a reduction in hospital admissions in the United Kingdom [16].

A novel SARS-CoV-2 variant, B.1.1.7, identified in September 2020 in the United Kingdom, has spread rapidly. Estimates suggest increased transmissibility and disease severity [17–20]. The lineage carries several mutations of immunologic significance, including N501Y located in the receptor-binding domain (RBD), a key neutralizing antibody target; deletions in the N-terminal domain at residues 69/70, associated with viral escape in the immunocompromised and S-gene target failure (SGTF) in PCR assays; and a deletion at residue 144 resulting in decreased monoclonal antibody binding [21].

Reinfection rates following natural infection have not been shown to be higher in studies using SGTF as a proxy for B.1.1.7, [20, 22] even though variably decreased sensitivity to neutralization by monoclonal antibodies, convalescent plasma and sera from vaccinated individuals has been observed in vitro for B.1.1.7 [23–34]. The Oxford-AstraZeneca trial showed good vaccine efficacy against sequencing-confirmed symptomatic B.1.1.7, despite evidence of decreased neutralizing titers but decreased efficacy for asymptomatic/unknown symptom infections [35]. Pfizer-BioNTech vaccine effectiveness in HCWs appears preserved despite increasing B.1.1.7 incidence in the United Kingdom; however, these studies have not specifically investigated cases of SGTF or sequencing-confirmed B.1.1.7 [8, 36].

We use an observational longitudinal cohort study of hospital HCWs to investigate and compare the protection from SARS-CoV-2 infection conferred by vaccination and prior infection (determined using anti-spike antibody status). Additionally, we estimate the protection provided by different vaccines, after 1 versus 2 doses and from infections with the B.1.1.7 variant confirmed by whole-genome sequencing.

## METHODS

### Setting

Oxford University Hospitals (OUH) offers symptomatic and asymptomatic SARS-CoV-2 testing to all staff at 4 hospitals and associated facilities in Oxfordshire, United Kingdom. SARS-CoV-2 PCR testing of nasal and oropharyngeal swabs for symptomatic (new persistent cough, fever ≥37.8°C, anosmia/ageusia) staff was offered from 27 March 2020. Asymptomatic HCWs were offered voluntary nasal and oropharyngeal swab PCR testing every 2 weeks and serological testing every 2 months from 23 April 2020, as previously described [2, 37, 38]. We report data to 28 February 2021. To minimize underascertainment of outcomes arising from staff leaving OUH’s employment, only those who participated in asymptomatic screening, symptomatic testing or vaccination from 1 September 2020 onward were included. We also performed a sensitivity analysis restricted to staff participating in asymptomatic screening or symptomatic testing from 1 September 2020. All staff working for the hospitals were eligible to participate.

### Laboratory Assays

Antibody status was determined using an anti-trimeric spike immunoglobulin G (IgG) enzyme linked immunosorbent assay (ELISA) [39] using an 8 million units threshold to determine antibody-positivity. PCR tests were performed by OUH using a range of PCR assays (see Supplementary materials). PCR-positive results from symptomatic community testing were also recorded. From 16 November 2020, OUH used the Thermo-Fisher TaqPath PCR assay as their first-line diagnostic assay, which includes orf1ab, S and N gene targets. As such SGTF indicative of the B.1.1.7 variant [20] could be identified, that is, orf1ab-positive/N-positive only. Oxford Nanopore sequencing was undertaken of all stored PCR-positive primary samples from 1 December 2020 onward to identify the infecting lineage (see Supplementary materials).

### Study Groups

Staff members were classified into 5 groups: (a) unvaccinated and consistently seronegative during follow-up; (b) unvaccinated and ever seropositive; (c) vaccinated once, always seronegative prior to vaccination; (d) vaccinated twice, always seronegative prior to first vaccination; (e) vaccinated (once or twice) and ever seropositive prior to first vaccination. The latter group were combined as relatively few staff were previously seropositive and vaccinated twice. Vaccinated groups were considered at-risk of infection >14 days after each vaccine dose (see Table 1 for further details of at-risk periods).

**Table 1.** Study Follow-Up Groups

Study Group	Description	Start of At-Risk Period	End of At-Risk Period, Study End or . . .
Unvaccinated, seronegative	Unvaccinated, consistently seronegative	The day of first negative antibody test	Positive PCR test
			First vaccination
			Positive antibody test
Unvaccinated, seropositive	Unvaccinated, and ever seropositive	>60 d after their first pre-vaccinated positive antibody test^a^	Positive PCR test
			First vaccination
Vaccinated once, previously seronegative	Vaccinated once, always seronegative prior to vaccination	>14 d after first vaccine dose	Positive PCR test
			Second vaccination
Vaccinated twice, previously seronegative	Vaccinated twice, always seronegative prior to vaccination	>14 d after second vaccine dose	Positive PCR test
Vaccinated, previously seropositive	Vaccinated (once or twice), and ever seropositive prior to first vaccination	>14 d after first vaccine dose	Positive PCR test

Abbreviation: PCR, polymerase chain reaction.

^a^To allow for any persistent RNA from the first infection and also requiring >60 days since the last positive PCR test. Those who were vaccinated without any prior antibody measurement were included in the previously seronegative follow-up groups.

Staff remained at risk of infection in each follow-up group until the earliest of the study end, first vaccination, second vaccination in previously seronegative HCWs, a positive PCR test, or for unvaccinated HCWs, a positive antibody test. Staff could transition from one group to another following seroconversion or vaccination after 60 or 14 days, respectively, disregarding any PCR-positive result during this crossover period, including the 14 days following a second vaccination for previously seronegative HCWs vaccinated twice.

The staff vaccination program began on 8 December 2020, starting with the Pfizer-BioNTech BNT162b2 vaccine, with the addition of the Oxford-AstraZeneca ChAdOx1 nCoV-19 vaccine from 4 January 2021. Some staff members received the ChAdOx1 nCoV-19 vaccine in clinical trials beginning 23 April 2020 and were included following unblinding.

### Outcomes

The main outcome was PCR-confirmed symptomatic SARS-CoV-2 infection. We also considered any PCR-positive result (ie, either symptomatic or asymptomatic). To assess the impact of the B.1.1.7 variant on (re)infection risk, we also analyzed PCR-positive results with and without SGTF, and those confirmed as B.1.1.7 on sequencing.

### Statistical Analysis

We used Poisson regression to model incidence of each outcome per day-at-risk by study group. We adjusted for calendar month, age, sex, self-reported ethnicity and staff occupational role, patient contact and working on a non-intensive care unit (ICU) ward caring for coronavirus disease 2019 (COVID-19) patients (previously shown to increase risk [37]) (details in Supplementary materials). We compared incidence in each follow-up group to unvaccinated seronegative HCWs, using incidence rate ratios (IRRs), such that 100*(1-IRR) is the percentage protection arising from being seropositive or vaccinated. We tested for heterogeneity by vaccine type. To assess timing of onset of protection we also fitted models in vaccinated individuals from day 1 postvaccination.

We used stacked Poisson regression to test for variation in the incidence of SGTF versus non-SGTF PCR-positive results, and B.1.1.7 versus non-B.1.1.7, considering only results from 1 December 2020 where S-gene PCR and sequencing were most complete.

We compared PCR cycle threshold (Ct) values between symptomatic and asymptomatic infections and by follow-up group using quantile regression.

### Ethics Statement

Deidentified data were obtained from the Infections in Oxfordshire Research Database which has generic Research Ethics Committee, Health Research Authority and Confidentiality Advisory Group approvals (19/SC/0403, 19/CAG/0144).

## RESULTS

**Figure 1.**
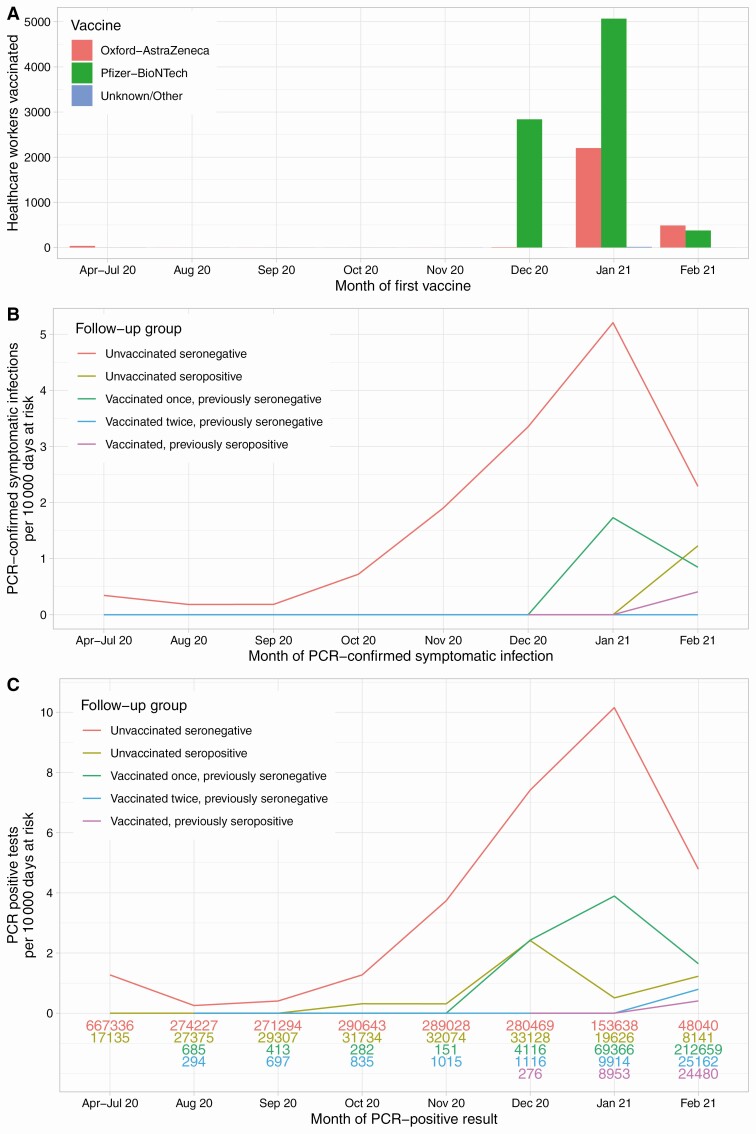
Vaccination timings (*A*) and observed incidence of symptomatic PCR-confirmed SARS-CoV-2 infection (*B*) and any PCR-positive result (*C*) by antibody and vaccine status. Some staff members received the Oxford-AstraZeneca vaccine in clinical trials beginning 23 April 2020 and were included following unblinding if in the active arm. Number of days at risk per month for each follow-up group is shown at the bottom of panel (*C*). Due to small numbers, rates are not plotted for vaccinated individuals prior to August 2020. Abbreviations: PCR, polymerase chain reaction; SARS-CoV-2, severe acute respiratory syndrome coronavirus 2.

In total, 13 109 individual HCWs contributed 2 835 260 person-days follow-up. Of these, 9765 (74%) were female, and the most common occupational roles were nurse (3579, 27%), doctor (1776, 14%), administrative staff (1688, 13%) and healthcare assistant (1263, 10%). The median (interquartile range [IQR]) age was 39 (30–50) years. Most HCWs were followed before vaccination: 10 513 HCWs were SARS-CoV-2 anti-spike IgG seronegative (2 274 675 person-days follow-up), and 1273 were seropositive (198 520 person-days). Most HCWs were vaccinated between December 2020 and January 2021 (Figure 1A); 8285 staff received Pfizer-BioNTech vaccine (1407 two doses) and 2738 Oxford-AstraZeneca vaccine (49 two doses). Eleven HCWs received another vaccine or could not recall the manufacturer. Staff could move between follow-up groups; in total there were 9711 and 940 previously seronegative HCWs followed after a first (289 134 person-days) and second (39 222 person-days) vaccine dose, respectively, and 974 (33 709 person-days) in the vaccinated previously seropositive group (108 of whom were vaccinated twice).

As previously reported [2], asymptomatic testing was less frequent in unvaccinated seropositive HCWs (127/10 000 person-days) than unvaccinated seronegative HCWs (185/10 000 person-days). Rates in previously seronegative and seropositive vaccinated staff were similar (163–169/10 000 person-days). Symptomatic testing followed a similar pattern (Supplementary Table 1).

### Incidence of PCR-Confirmed Symptomatic SARS-CoV-2 Infection

**Figure 2.**
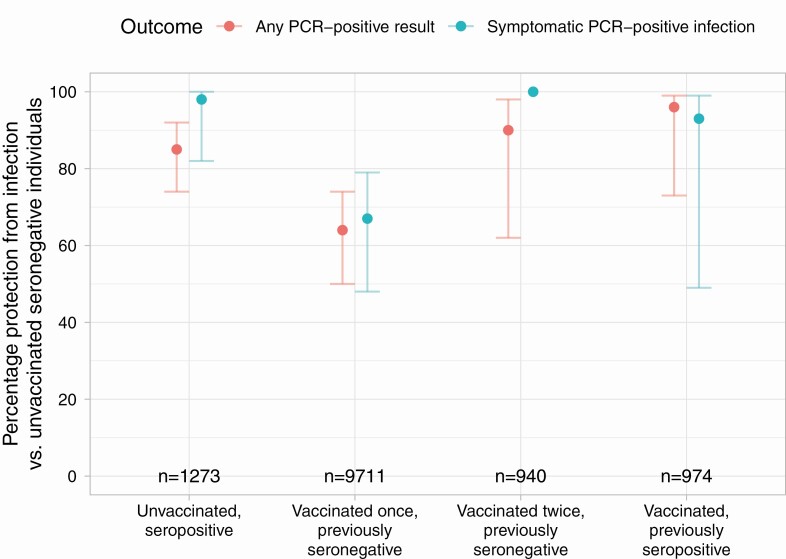
Protection from infection by antibody and vaccination status, compared with unvaccinated seronegative individuals. Number of HCWs in each follow-up group is shown; 95% confidence intervals are plotted, except for previously seronegative HCWs vaccinated twice who had no symptomatic PCR confirmed infections. Abbreviations: HCW, healthcare worker; PCR, polymerase chain reaction.

PCR-confirmed symptomatic SARS-CoV-2 infection in HCWs peaked in December 2020 and January 2021, similarly to local community-based infection rates [40] (Figure 1B, Table 2 and Supplementary Table 2). Also, 294 unvaccinated seronegative HCWs were infected, 1 unvaccinated seropositive HCW and 32 vaccinated HCWs > 14 days post first vaccine (1 previously seropositive). Compared to unvaccinated seronegative HCWs who had the highest rates of infection, incidence was 98% lower in unvaccinated seropositive HCWs (adjusted IRR [aIRR] 0.02 [95% confidence interval {CI} < .01–.18; *P* < .001]), and 67% lower following a first dose in previously seronegative HCWs (aIRR = 0.33 [95% CI .21–.52; *P* < .001]), with no symptomatic infections seen following a second dose (Figure 2). Incidence was also 93% lower in vaccinated previously seropositive HCWs (aIRR = 0.07 [95% CI .01–.51; *P* = .009]). Incidence was higher following a first vaccination than in seropositive HCWs (*P* = .01), but there was no evidence of difference between seropositive HCWs and following a second vaccination (*P* = .96). Independently of vaccination and antibody status, rates of infection were higher in staff caring for SARS-CoV-2-infected patients, in nurses and healthcare assistants, and in staff of Asian ethnicity (Table 2). Results from a sensitivity analysis restricting to only those participating in testing from 1 September 2020 were similar (n = 11,758 HCWs, Supplementary Table 3).

**Table 2.** Adjusted Incidence Rate Ratios (IRRs) for Symptomatic PCR-Confirmed SARS-CoV-2 Infection and Any PCR-Positive Result (Symptomatic or Asymptomatic) by Antibody and Vaccine Status

Variable		Symptomatic PCR-confirmed Infection			Any PCR-positive Result		
		Adjusted IRR	95% CI	*P* value	Adjusted IRR	95% CI	*P* value
Age	Age, per 10 y increase	0.92	.84–1.02	.10	0.99	.99–1.00	.07
Sex	Female (reference)	1.00			1.00		
	Male	1.11	.85–1.44	.46	1.08	.90–1.31	.41
Patient facing role	No (reference)	1.00			1.00		
	Yes	1.06	.76–1.49	.72	1.13	.90–1.42	.29
Covid ward	Not working in Covid ward	1.00			1.00		
	Working in non-ICU Covid ward	1.57	1.11–2.21	.01	1.53	1.21–1.94	<.001
Month	April—July 2020 (reference)	1.00			1.00		
	August 2020	0.51	.19–1.33	.17	0.20	.09–0.42	<.001
	September 2020	0.51	.20–1.35	.18	0.31	.17–.58	<.001
	October 2020	2.02	1.13–3.63	.02	1.01	.69–1.48	.96
	November 2020	5.34	3.30–8.62	<.001	2.92	2.20–3.87	<.001
	December 2020	9.23	5.89–14.50	<.001	5.91	4.60–7.59	<.001
	January 2021	14.60	9.24–23.00	<.001	7.93	6.10–10.30	<.001
	February 2021	7.10	3.89–13.00	<.001	3.72	2.54–5.46	<.001
Follow up group	Unvaccinated seronegative (reference)	1.00			1.00		
	Unvaccinated seropositive	0.02	<01–.18	<.001	0.15	.08–.26	<.001
	Vaccinated once, previously seronegative	0.33	.21–.52	<.001	0.36	.26–.50	<.001
	Vaccinated twice, previously seronegative	No events			0.10	.02–.38	<.001
	Vaccinated, previously seropositive	0.07	.01–0.51	.009	0.04	.01–.27	.001
Ethnic group	White (reference)	1.00			1.00		
	Asian	1.90	1.47–2.46	<.001	1.59	1.32–1.91	<.001
	Black	1.09	.62–1.91	.78	1.25	.88–1.78	.21
	Other	1.31	.87–1.97	.20	1.23	.93–1.62	.16
Role	Other (reference)	1.00			1.00		
	Junior doctor	1.35	.86–2.12	.20	1.10	.78–1.54	.59
	Senior doctor (consultant)	0.50	.24–1.05	.07	0.60	.38–.95	.03
	Healthcare assistant	1.71	1.18–2.47	.005	1.82	1.42–2.34	<.001
	Nurse	1.50	1.11–2.03	.009	1.49	1.21–1.83	<.001
	Physio-, occupational or speech/language therapist	0.65	.26–1.61	.35	1.17	.73–1.88	.51
	Porter, domestic staff	1.05	.48–2.29	.91	1.37	.84–2.22	.20
	Administrator	0.99	.64–1.52	.96	1.18	.89–1.57	.25

Event counts, follow-up, and unadjusted IRRs are provided in Supplementary Table 2.

Abbreviations: CI, confidence interval; ICU, intensive care unit; PCR, polymerase chain reaction; SARS-CoV-2, severe acute respiratory syndrome coronavirus 2.

Thirty-eight unvaccinated seronegative HCWs attended hospital within −2 to + 28 days of a SARS-CoV-2 PCR-positive result (14.2/million person-days); of these, 27 had a COVID-19 primary diagnostic code, and 16 required admission for COVID-19. Two previously seronegative vaccinated HCWs required hospital review (6.9/million person-days); however, neither required admission. No HCW vaccinated twice or unvaccinated seropositive HCW required hospital review or admission.

### Incidence of Any PCR-Confirmed Symptomatic or Asymptomatic SARS-CoV-2 Infection

Rates of any PCR-positive result, irrespective of symptoms, were highest in unvaccinated seronegative HCWs (635 cases), with 85% lower incidence in unvaccinated seropositive HCWs (12 cases, aIRR = 0.15 [95% CI .08–.26, *P* < .001]). Incidence was reduced by 64% in seronegative HCWs following first vaccination (64 cases, aIRR = 0.36 [95% CI .26–.50; *P* < .001]) and 90% following second vaccination (2 cases, aIRR = 0.10 [95% CI .02–.38; *P* < .001]) (Figures 1C and 2, Table 2 and Supplementary Table 2). Incidence was also 96% lower in vaccinated previously seropositive HCWs (1 case, aIRR = 0.04 [95% CI .01–.27; *P* = .001]). As seen above for symptomatic infection, protection from any PCR positive result irrespective of symptoms was lower following first vaccination than if seropositive (*P* = .006) but with no evidence of difference between seropositivity and second vaccination (*P* = .59).

### PCR-Positive Results Following Vaccination

**Figure 3.**
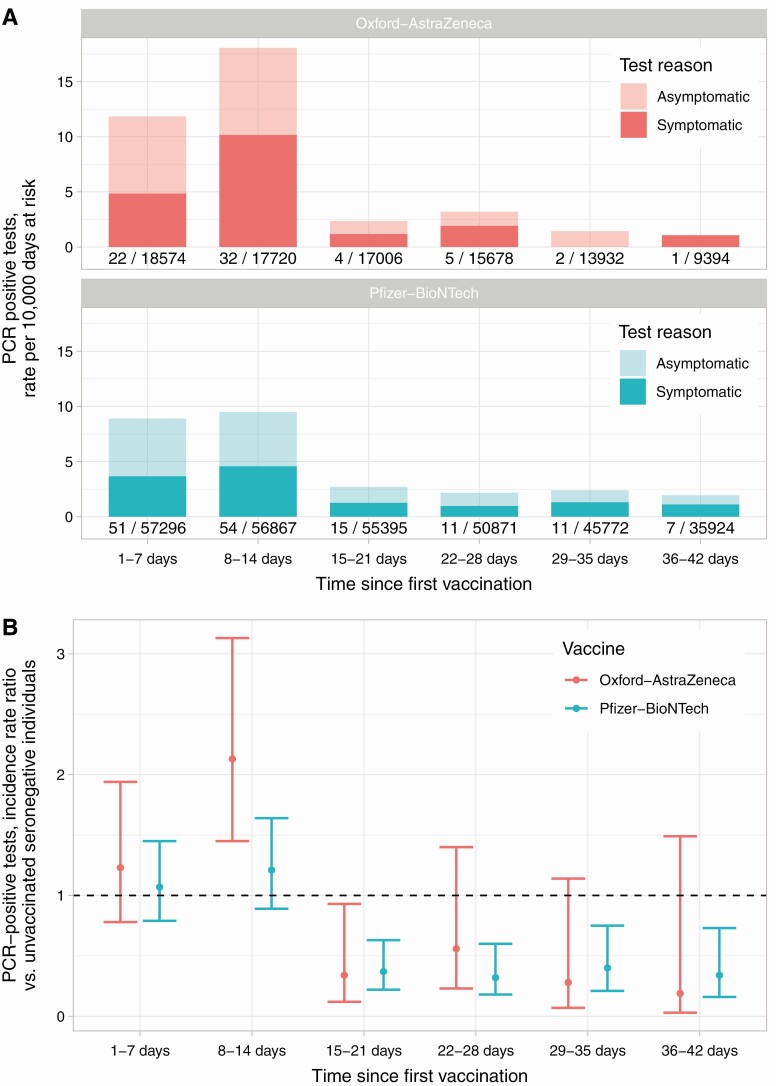
PCR-positive results following first vaccination. *A*, Observed rates of symptomatic and asymptomatic PCR-positive results; counts and days at risk plotted under each bar. *B*, Relative incidence of PCR-positive results by vaccine and days since first vaccine compared to rates in unvaccinated seronegative HCWs. For both plots follow-up is censored if a second vaccination was given. Abbreviations: HCW, healthcare worker; PCR, polymerase chain reaction.

The incidence of PCR-positive results fell from >14 days after the first vaccination for both the Pfizer-BioNTech and Oxford-AstraZeneca vaccines, with similar levels of protection seen up to 42 days postvaccine (Figure 3). There was an unexpected rise in incidence above baseline levels in the first two weeks following vaccination, which remained to some extent after adjustment (Figure 3B). Considering efficacy against any PCR-positive result >14 days post first dose, there was no evidence of a difference by vaccine type following the first (heterogeneity *P* = .33) or second (*P* = .16) dose. Similarly, there was no evidence of difference in PCR-confirmed symptomatic SARS-CoV-2 infection (*P* = .21 and *P* > .99, respectively).

### Impact of Antibody Status and Vaccination on Viral Loads

**Figure 4.**
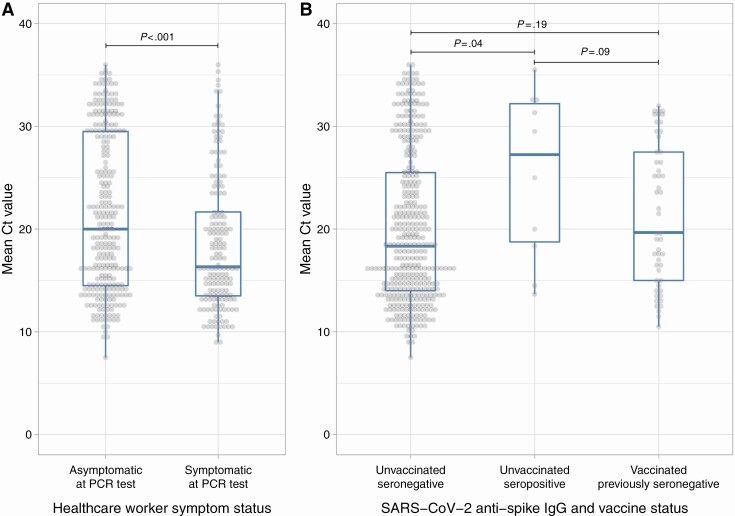
Relationship between SARS-CoV-2 PCR cycle threshold (Ct) values and symptoms (*A*), antibody and vaccine status (*B*). Ct values were available for HCWs tested using the Thermo-Fisher TaqPath assay from 16 November 2020 onwards, n = 423. Mean per sample Ct value across all detected targets is shown. For panel *A*, Kruskal-Wallis *P* < .001; for panel *B*, Kruskal-Wallis *P* = .06, Wilcoxon rank sum test *P* values are shown between categories. Abbreviations: HCW, healthcare worker; IgG, immunoglobulin G; PCR, polymerase chain reaction; SARS-CoV-2, severe acute respiratory syndrome coronavirus 2.

Viral loads were higher, that is, Ct values lower, in symptomatic infections (median [IQR] Ct: 16.3 [IQR 13.5–21.7]) compared to asymptomatic screening (Ct: 20 [IQR 14.5–29.5]) (Figure 4A, Kruskal-Wallis *P* < .001). Unvaccinated seronegative HCWs had the highest viral loads (Ct: 18.3 [IQR 14.0–25.5]), followed by vaccinated previously seronegative HCWs (Ct: 19.7 [IQR 15.0–27.5]); unvaccinated seropositive HCWs had the lowest viral loads (Ct: 27.2 [IQR 18.8–32.2]) (Figure 4B, overall *P* = .06). Combining symptom status and prior-antibody/vaccine status, there was a trend toward prevaccine seropositivity and vaccination independently decreasing viral loads, reflected in Ct value increases of 5.7 (95% CI −.9, +13.2) and 2.7 (−.5, +6.8), respectively (Supplementary Table 4).

### Incidence of SGTF and B.1.1.7 SARS-CoV-2 Infection

**Figure 5.**
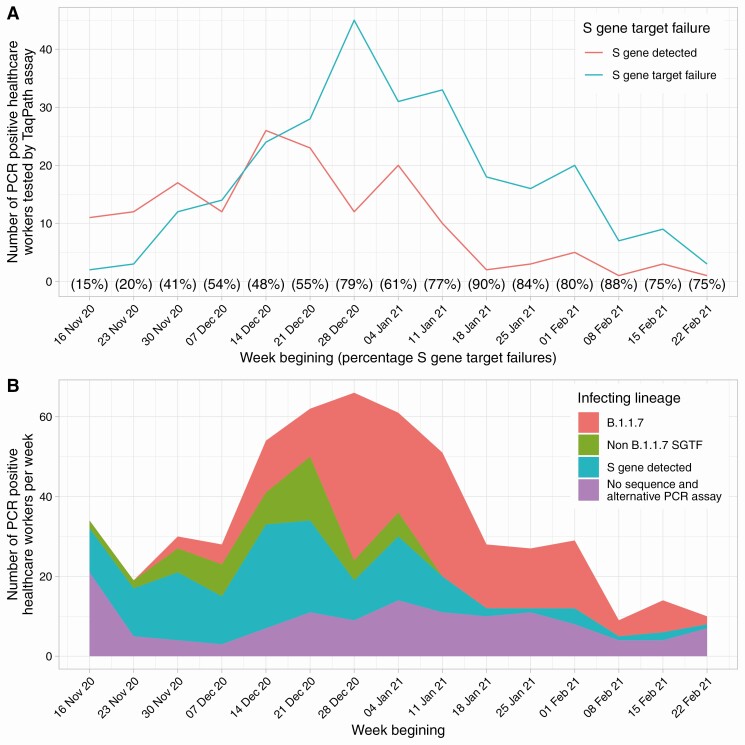
Incidence of SGTF (*A*) and B.1.1.7 (*B*) infection by week of testing. From 16 November 2020 onward samples from HCWs were routinely processed using the Thermo-Fisher TaqPath assay allowing SGTF to be identified, shown in panel *A*. Sequencing was undertaken of samples processed on other assays as well, hence the larger total in panel *B*. Abbreviations: HCW, healthcare worker; PCR, polymerase chain reaction; SGTF, S gene target failure.

From 1 December 2020, SGTF status was determined for 390/463 (84%) PCR-positives (with the majority of remaining positive tests undertaken in the community); 258/390 (66%) had SGTF. SGTF accounted for 15% of positive PCR results in mid-November 2020, rising to 90% in the second half of January 2021, before declining again (Figure 5A). There was no evidence that SGTF changed the extent of protection against any PCR-positive infection in unvaccinated seropositive HCWs (aIRR vs non-SGTF, 0.43, [95% CI .12–1.52; *P* = .19]) or previously seronegative HCWs after a first vaccine (1.13 [95% CI .48–2.63; *P* = .78]).

We used viral whole-genome sequencing to determine infecting lineages from 1 December 2020 onward (Supplementary Table 5): 343/463 (74%) were successfully sequenced, 193/343(56%) were B.1.1.7, an additional 19/463 (4%) were not sequenced but S-gene positive (ie, unlikely B.1.1.7) (Figure 5B). There was no evidence that B.1.1.7 changed the extent of protection from any-PCR positive infection in those who were seropositive (aIRR vs non-B.1.1.7 = 0.40 [95% CI .10–1.64; *P* = .20]) or following a first vaccine dose (aIRR = 1.84 [95% CI .75–4.49; *P* = .18). Seventeen percent of SGTF was due to a lineage other than B.1.1.7. No other variants of concern (B.1.1.7 with E484K, B.1.351 or P.1) were identified in participating HCWs, in an at-risk period.

## Discussion

In this longitudinal cohort study of HCWs receiving Pfizer-BioNTech and Oxford-AstraZeneca vaccines, vaccination reduced the incidence of PCR-positive symptomatic SARS-CoV-2 infection, with 2 doses providing similar levels of protection to natural immunity. No symptomatic infections were seen following two vaccine doses and there was a 98% reduction in symptomatic infections in unvaccinated seropositive HCWs. Protection was still afforded >14 days after a single vaccine dose, albeit at lower levels (67% reduction). No vaccinated HCW required hospital admission. Furthermore, vaccination reduced the incidence of any PCR-positivity by 64% and 90% >14 days post-first and second vaccine dose, respectively, compared to an 85% reduction post-natural infection. This suggests that both vaccination and previous infection are also likely to reduce transmission. Additionally, there was a trend toward reduced viral loads in reinfected individuals compared to infected seronegative HCWs, with a smaller observed reduction postvaccination.

The comparable protection offered by seropositivity to 2 doses of vaccine suggests that the immunoassay used provides an accurate correlate of immunity, which could potentially be used to support individualized relaxation of societal restrictions. Furthermore, where vaccine supplies are limited prioritizing seronegative infection-naïve individuals may be appropriate.

Protection following 2 vaccine doses was comparable to other real-world studies [6, 8]. Protection following a single dose was toward the lower range of previous reports, potentially reflecting occupational exposure in HCWs. Although an unexpected rise in incidence was seen in the first 2 weeks postvaccination, this time period was excluded from effectiveness calculations. Possible explanations include increased ascertainment of asymptomatic infection due to vaccine-related symptoms leading to testing, behavior change, acquisition at vaccination facilities, or staff attending for vaccination prompted by high levels of exposure to infected colleagues or patients. A similar rise in incidence was noted in the Israeli mass vaccination program, attributed to behavior change post-vaccination [11, 12].

Immunity induced by natural infection and vaccination was robust to lineage, including cases confirmed to be B.1.1.7 by whole-genome sequencing, at least within the power of the study. Sequencing was important to confirm the lineage of SGTF cases: although >99% del69-70 sequences from Southeast England were due to B.1.1.7 over this period [20], locally 17% of SGTF was due to other non-B.1.1.7 lineages. Assuming all SGTF is B.1.1.7 risks misestimating the impact of this lineage on natural and vaccine-induced immunity. This reinforces the need to understand local genomic epidemiology and the reliability of SGTF as a proxy for B.1.1.7 over time. Our results are comparable with the Oxford-AstraZeneca analysis of vaccine efficacy against B.1.1.7 based on a relatively low proportion of successfully sequenced cases (179/499, 36%) and no documentation of SGTF status [35], compared to this study, where PCR and WGS confirmed SGTF/lineage status in 78% cases.

One important finding is that despite universal use of personal protective equipment (gloves, plastic aprons, surgical marks for all patient care and FFP3 masks, gowns and eye protection for aerosol generating procedures), social distancing, and use of surgical masks throughout all areas of the hospital, staff working in COVID-19 wards remained at higher risk of SARS-CoV-2 infection independent of vaccine and antibody status. Possible explanations include acquisition from patients with or without subsequent amplification by staff-to-staff spread. Nurses, healthcare assistants, and Asian staff were also at higher risk of infection, possibly reflecting both hospital and community-based exposures as we have discussed previously [37].

One study limitation is that staff working in roles more likely to be exposed to SARS-CoV-2 were initially prioritized for vaccination; these staff were also at the greatest risk of occupationally-acquired SARS-CoV-2 infection. We adjusted for this by including working in a COVID-19 ward and staff roles, but incomplete adjustment could lead to underestimation of vaccine efficacy. Similarly, vaccinated staff were potentially more likely to be current employees than unvaccinated staff; if unvaccinated seronegative staff left employment this would potentially lead to underascertainment of infection in this group. We address this by only including staff using testing and/or vaccination services in the last 6 months of the study. Testing rates were lower in seropositive HCWs and to a lesser extent following vaccination, leading to underascertainment of PCR-positive results in these groups; however, we have previously demonstrated the impact of this is relatively small [2]. Other limitations include limited power to detect differences in efficacy between vaccines. We were also unable to sequence all PCR-positives, in particular because those with higher Ct values are less likely to generate high-quality sequences, and some samples were not stored, including those processed by community testing facilities. Similar studies will be needed to assess the vaccine effectiveness against other, novel emerging SARS-CoV-2 lineages. Finally, this is a study of HCWs of working age, so findings may not generalize to other settings.

In summary, by pooling data from unvaccinated and Pfizer-BioNTech and AstraZeneca vaccinated HCWs, we show that natural infection resulting in detectable anti-spike antibodies and 2 doses of vaccine both provide robust protection against SARS-CoV-2 infection, including against the B.1.1.7 variant of concern.

## Supplementary Data

Supplementary materials are available at *Clinical Infectious Diseases* online. Consisting of data provided by the authors to benefit the reader, the posted materials are not copyedited and are the sole responsibility of the authors, so questions or comments should be addressed to the corresponding author.

ciab608_suppl_Supplementary_Data

## Notes

***Acknowledgments.*** The authors would like to thank all OUH staff who participated in the staff testing program, and the staff and medical students who ran the program. This work uses data provided by healthcare workers and collected by the UK’s National Health Service as part of their care and support. The authors thank all the people of Oxfordshire who contribute to the Infections in Oxfordshire Research Database. Research Database Team: L Butcher, H Boseley, C Crichton, DW Crook, DW Eyre, O Freeman, J Gearing (community), R Harrington, K Jeffery, M Landray, A Pal, TEA Peto, TP Quan, J Robinson (community), J Sellors, B Shine, AS Walker, D Waller. Patient and Public Panel: G Blower, C Mancey, P McLoughlin, B Nichols. The data sets analyzed during the current study are not publicly available as they contain personal data but are available from the Infections in Oxfordshire Research Database (https://oxfordbrc.nihr.ac.uk/research-themes-overview/antimicrobial-resistance-and-modernising-microbiology/infections-in-oxfordshire-research-database-iord/), subject to an application and research proposal meeting the ethical and governance requirements of the Database. Sequence data generated during the study are available from the European Nucleotide Archive under study accession number PRJEB43319.

Oxford University Hospitals Staff Testing Group (Contributors): University of Oxford Medical School staff testing team (University of Oxford, Oxford, UK): Afrah Shibu, Aisling Curtis, Alexandra Mighiu, Ali Manji, Andrey Nezhentsev, Arun Somanathan, Beinn Khulusi, Ben Holloway, Caitlin Rigler, Charis Virgo, Charlotte Fields, Charlotte Lee, Elizabeth Daly, Elizabeth Hatton, Esme Weeks, Euan McGivern, Greta Economides, Hannah Fuchs, Harry Jackson-Smith, Heather Tong, Helen Callard, Helen Clay, Henrietta Davies, Isaac Jarratt Barnham, Ishta Sharma, Jack Wilson, Jocelyn Ward, Joseph Cutteridge, Julia Kotowska, Kirsten Lee, Krupa Ravi, Laura Wilkins, Lottie Cansdale, Lucy Bland, Luiza Farache Trajano, Magdalena Chmura, Maria Lucey, Maria Pikoula, Meirian Evans, Molly Abbott, Morwenna Tamblyn, Oriane Grant, Rebecca Conway-Jones, Ross Toward, Roxanna Abhari, Ruby Wolman, Sara Hosseinzadeh, Sarah Thomas, Tara Madsen, Thomas H Foord, Thomas Johnson, Vimukthi Perera, Zamin Shabir

University of Oxford staff testing team (University of Oxford, Oxford, UK): Thomas Christott, George Doherty, Philip W Fowler, Fredrik Karpe, James Kavanagh, Lucas Martins Ferreira, Matt J Neville, Hayleah Pickford, Donal Skelly, Jeremy Swann, Sarah Cameron, Phoebe Tamblin-Hopper, Magda Wolna, Rachael Brown, Denis Volk, Fan Yang-Turner, Alison Vaughan

Oxford University Hospitals staff testing team (Oxford University Hospitals NHS Foundation Trust, Oxford, UK): Adrian Bialek, Alison Whitty, Annie Westlake, Barbara Wozniak, Bryony Butler, Claudio Ferreira, Danielle Russell, Dawn Pether, Elaine Lawson, Eleanor Ross, Eleni Fragkouli, Elizabeth Sims, Emma Mortimore, Geraldine Shaw, Harriet Mullins, Harriett Caroll, Jane Phillips, Jenny Brown, Jess Ponting, Justyna Szczurkowska, Kim Vilca, Kitty Norris, Louise Holland, Michael Luciw, Michelle Gates, Michelle Layton, Nicola Antonucci, Noemi Bodo, Rebecca Millard, Sara Lyden, Sarah Young, Simran Barot, Vanessa Cox, Victoria Wharton, Zoe Thompson

Oxford University Hospitals microbiology laboratory (Oxford University Hospitals NHS Foundation Trust, Oxford, UK): Anne Baby, Jasmine Bastable, Kathryn Cann, Reena Chohan, Josie Clarke, Gabriel Cogorno, Samantha Cordy, Georgina Coward, David Crawford-Jones, Sean Crawley, Jack Dobson, Bronte Drummond, Laura Dunn, Caleb Edwin, Simon Evans, Mohamad Fadzillah, Jessica Gentry, Sarah Hill, Laura Hobden, Nurul Huda, Gemma Innes, Scott Jarvis, Gerald Jesuthasan, Emma Jones, Anita Justice, Elizabeth Kalimeris, Richard Kirton, Nakiah Lashley, Sophie Mason, Alexander Mobbs, Ahila Murugathasan, Eleanor Mustoe, Gospel Ngoke, Sarah Oakley, Oliver O’Sullivan, Kimberley Odwin, Jack Oliver, Freyja Pattrick, Claudia Pereira, Simon Perry, Tom Potter, Alexander Prentice, Sophie Ramage, Athena Sanders, Kellyanne Savage, Katherine Shimell, Robin Terry, Emma Thornton, Susan Wareing, Annie Welbourne, Maddison Wheatley

Oxford University Hospitals Infection, Prevention and Control team (Oxford University Hospitals NHS Foundation Trust, Oxford, UK): Lisa Butcher, Gabriella D’Amato, Ruth Moroney, Gemma Pill, Lydia Rylance-Knight, Claire Sutton, Claudia Salvagno, Merline Tabirao, Sarah Wright.

***Financial Support.*** This work was supported by the UK Government’s Department of Health and Social Care. This work was also supported by the National Institute for Health Research Health Protection Research Unit (NIHR HPRU) in Healthcare Associated Infections and Antimicrobial Resistance at Oxford University in partnership with Public Health England (PHE) (grant number NIHR200915), the NIHR Biomedical Research Centre, Oxford, and benefactions from the Huo Family Foundation and Andrew Spokes. The views expressed in this publication are those of the authors and not necessarily those of the NHS, the National Institute for Health Research, the Department of Health or Public Health England. This study is affiliated with Public Health England’s SARS-CoV-2 Immunity & Reinfection EvaluatioN (SIREN) study.

D. W. E. is a Robertson Foundation Fellow and an NIHR Oxford BRC Senior Fellow. S. F. L. is a Wellcome Trust Clinical Research Fellow. D. I. S. is supported by the Medical Research Council (grant number MR/N00065X/1). P. C. M. holds a Wellcome Intermediate Fellowship (grant number 110110/Z/15/Z). B. D. M. is supported by the SGC, a registered charity (grant number 1097737) that receives funds from AbbVie, Bayer Pharma AG, Boehringer Ingelheim, Canada Foundation for Innovation, Eshelman Institute for Innovation, Genome Canada through Ontario Genomics Institute (grant number OGI-055), Innovative Medicines Initiative (EU/EFPIA) (ULTRA-DD grant number 115766), Janssen, Merck KGaA, Darmstadt, Germany, MSD, Novartis Pharma AG, Pfizer, Sao Paulo Research Foundation-FAPESP, Takeda, and Wellcome. B. D. M. is also supported by the Kennedy Trust for Rheumatology Research. G. S. is a Wellcome Trust Senior Investigator and acknowledges funding from the Schmidt Foundation. T. M. W. is a Wellcome Trust Clinical Career Development Fellow (grant number 214560/Z/18/Z). A. S. W. is an NIHR Senior Investigator.

***Potential conflicts of interest.*** D. W. E. declares lecture fees from Gilead, outside the submitted work. R. J. C. is a founder, shareholder, and consultant to MIROBio, outside the submitted work. All other authors report no potential conflicts.
